# Gender differences in reaction to Covid19 in people with Autism Spectrum Disorder

**DOI:** 10.1192/j.eurpsy.2022.1354

**Published:** 2022-09-01

**Authors:** R.J. Van Der Gaag, P. Van Wijngaarden-Cremers

**Affiliations:** 1 Radboud University Medical Centre, Psychiatry, Nijmegen, Netherlands; 2 Stradina University, Psychosomatics And Psychotherapy, Riga, Latvia

**Keywords:** autism, confinement, covid 19, gender

## Abstract

**Introduction:**

The Covid 19 pandemic has had an enormous psychological impact in which women were mentally more affected than men (Berthelot et al 2020 - Liu et al 2020). More over it exacerbated symptoms and suffering in individuals with psychiatric disorders. The question is how did it affect individuals with autism (ASD). And if so, did it affect women and girls more that men and boys or differently.

**Objectives:**

To evaluate the impact of the covid 19 pandemic and the subsequent lockdown in patients with ASD

**Methods:**

a combination of literature review and assessement of the population of individuals with autism spectrum disorders in a large specialized unit for individuals with ASD

**Results:**

No seperate reports on individuals with ASD were found in the recent literature. Obviously the pandemic and confinement had great impact on individuals with intellectual disabilities amongst whom many individuals with comorbid developmental disorders as ADHD and/or Autism (e.g.Palacio-Ortiz et al 2020 - Guessoum et al 2020) In our population some remarkable outcomes were noted in the sense that a substantial part of the population with autism had positive experiences: e.g. clear social rules / less contact. Males enjoyed more online contact and games / experienced less loneliness - In females we found significantly more depressive symptoms, anxiety and suicidal behaviour.

**Conclusions:**

Covid 19 has had a great psychological impact with marked gender differences. Remarkably in ASD men enjoyed some of the benefits of the confinement. In women with ASD the emotional impact was significantly higher.

**Disclosure:**

No significant relationships.

